# Medical Opinion and Movement

**Published:** 1909-10-16

**Authors:** 


### Medical Opinion and Movement.

In an article in the *Presse Médicale* Guibé draws attention to a curious morbid entity which has hitherto been little recognised—namely, a varicose condition of the renal papillæ as a cause of Hæmaturia. Fenwick first pointed out in 1904 the presence of angioma or nævus of the renal papillæ as a cause of hæmaturia. Other lesions, such as varicose veins of the legs, varicocele, and hæmorrhoids, are frequently associated with the condition, indicating a general varicose diathesis. In regard to varicocele, the author suggests that, as the pampiniform plexus terminates directly in the renal vein close to the kidney, it is probable that the varicose dilatation may invade the renal vein and so extend to its ramifications in the kidney. Anatomically the papillæ may show in some cases a varicose condition in the vessels of the mucous membrane visible to the naked eye. In others the lesions are only detected microscopically. Usually there is no evidence of nephritis or other abnormal condition. The chief and often only symptom is hæmaturia, which is sometimes profuse. It may be continuous or intermittent, returning at long intervals. There is usually no pain or other symptom, except, perhaps, somewhat frequent micturition. There is little to be done in the way of treatment, but in severe cases where rest, calcium salts, or other hæmostatics are ineffective, surgical intervention may be necessary.

---

Some interesting researches have been carried out by Dr. A. Gramegna upon the relations between the pressure of cerebro-spinal fluid and the general blood-pressure. In normal individuals with an arterial tension of 120 to 140 millimetres, there is a pressure of the cerebro-spinal fluid of 18 to 22 millimetres, and conditions which cause an increase or diminution of the one tend to produce a change in the other in the same sense. The hypotensive effect of hæmorrhage or shock upon the cerebro-spinal fluid is already well known. Lumbar puncture in patients with arteriosclerosis will cause a fall in blood-pressure lasting for several hours or even days, and amounting in some cases to 50 millimetres or more. The author divides the conditions which may occur into three classes. The first class comprises patients suffering from neurasthenia, tabetic cachexia with arterial and cerebro-spinal hypotension. In one case of the latter condition the blood-pressure was 90 millimetres and that of the cerebro-spinal fluid 4 millimetres. The second class, the arteriosclerotic, has arterial and cerebro-spinal hypertension, and the third condition shows cerebro-spinal hypertension combined with arterial hypotension, such as is found in cases of cerebral tumour and meningitis. This last condition, therefore, indicates some organic lesion of the central nervous system. In regard to the first class of case the author suggests that certain forms of neurasthenia may be connected with this hypotension of the cerebro-spinal fluid, and that the good effects of faradisation and hydrotherapy may be due to an elevation of the pressure.

At the recent International Congress of Medicine at Budapest, Dr. M. V. Artom di Saint Agnese, of Rome, gave an account of his researches into the Ætiology of Osteomalacia, by which he confirms the previous work of Arcangeli, Levi, and others, definitely indicating the microbic origin of this disease. He claims to have obtained in eight out of nine cases of osteomalacia positive bacteriological results, having in each case isolated the diplococcus osteomalaciæ in pure culture. Moreover, by introducing under the skin of the back fragments of bone taken from a woman suffering from the disease he has succeeded in reproducing the disease in white rats. The inoculations were made on seven rats, and all became ill and died within the following three months. Pregnant female rats developed much more serious bony lesions than the males. There were marked changes in the conformation of the bony tissue as shown by the $x$-rays and by the microscope, and several spontaneous fractures occurred. Other animals were infected with the diplococcus obtained from those of the first series and also developed marked symptoms of osteomalacia. In collaboration with Dr. Arcangeli the author has prepared a vaccine by attenuation of the cultures with heat, and has had some very satisfactory results in the treatment of several cases of osteomalacia, even of long standing. He has also obtained a micrococcus from cases of rickets in children which closely resembles the diplococcus of puerperal osteomalacia, and which inoculated into rats produces a condition in all respects similar to that resulting from inoculation with the diplococcus osteomalaciæ, so that the author is convinced of the infective nature of rickets and of the identity of the two diseases, osteomalacia and rickets.

---

It is only in recent years and since the discovery of the internal secretions of such glands as the Suprarenals and Pancreas that efforts have been made to grasp the pathological possibilities of these organs, their involvement in general diseases, and the effects of lesions of these organs upon the general organism. Dr. Morichau-Beauchant, of Poitiers, makes an interesting contribution to *Le Progrès Medical* on suprarenal insufficiency in acute infections, in which he endeavours to demonstrate that there is a syndrome of symptoms which frequently appear in course of some of the infectious fevers, especially diphtheria, typhoid, and scarlatina, and which is attributable to a deficiency in the functioning powers of the suprarenals resulting from involvement of these organs in the disease. This syndrome is characterised chiefly by a condition of profound asthenia, accompanied by a considerable fall in blood-pressure, a small, rapid pulse, often irregular, a tendency to vertigo and syncope, and the suprarenal "white line" first described by Lugent and produced in a similar manner to the "tache cerebrale" of meningitis. Gastro-intestinal disturbances, vomiting, diarrhœa, and colic pains are frequent, and the urine is generally diminished in quantity and in nitrogenous contents. Autopsies on patients who have succumbed in this condition have shown lesions of the suprarenals of varying nature, including congestion, hæmorrhages, cavitation, and cellular degeneration. Finally, the author pleads for the treatment of this condition of suprarenal insufficiency by opotherapy or the administration of the suprarenal gland or adrenalin. The treatment may be carried out by giving the fresh gland or prepared dry extracts of it, or by giving the adrenalin solution by the mouth or by subcutaneous solution; 10 to 20 drops of 1 in 1,000 solution may be given five or six times a day. Frequent small doses are best as the effect is transitory.

---

Professor G. A. Buckmaster, in his introductory lecture at the Royal Veterinary College, Great College Street, last week, said that it was in the matter of the supervision of the milk and meat supply that an extended sphere of utility lay before veterinary surgeons, for a trained veterinary surgeon was the proper person who should, by reason of special knowledge, be qualified to give an opinion as to whether an animal intended to be slaughtered for food was healthy or not, and in any efficient system of meat inspection all animals intended for food should be passed by an examination which was conducted before as well as after slaughter. He advocated the creation of a special diploma for the veterinary surgeon who had made a special and extended study of these subjects, and contended that the whole of the research work of both the medical and veterinary professions should be financed by the State.

---

In an article in the *Therapeutic Gazette*, Benedict discusses the value of Phenolphthalein from the therapeutic standpoint. It has long been known as a useful laxative, though it has but little effect when administered in a single dose with the object of clearing out the bowels. Besides this laxative action, the drug also has cholagogic properties and produces a free flow of bile, at the same time exerting a certain bactericidal action and checking local bacterial processes in the gall-bladder and biliary passages. It is not, however, a solvent of gall-stones. Phenolphthalein produces its effects by direct irritation, peristalsis being consequently increased. It is not toxic, however, except when taken in large doses. By its use Indican and Indol are seemingly made to disappear from the urine and fæces respectively, though not more rapidly than can be accounted for by its purgative action alone. It is best when prescribing it as a laxative to give from half to three-quarters of a grain of phenolphthalein three times a day for a week, the effect being graded by varying the frequency of the dose. The drug is eliminated in the urine, and it is possible to titrate the acidity of urine from its own content of this indicator. Actual colouration of the urine is rare except when decomposition is present as the result of bacterial processes, or after the administration of large doses of alkali. The drug has little tendency to interfere with the ordinary urinary tests except those for peptone and sugar, or when an alkaline solution of copper is used. Some diagnostic confusion may, however, arise when vessels are used in which traces of alkali or soap still remain. That it has a certain antiseptic action would seem proved from the fact that the author has found it possible to prevent by its use obvious decomposition of urine for a period of more than a week.

---

There appears in the *American Journal of the Medical Sciences* an article from the pen of Clarke, in which he gives an account of his researches into the effects of certain so-called milk-modifiers on the gastric digestion of infants and the conclusions which he draws therefrom. He finds that the motility of the infant stomach varies inversely as the concentration of the food, and that consequently the more dilute the food is, the more frequently can the infant be fed. No effect on the acidity of the gastric contents is exerted by the presence of lime-water in the stomach, since the neutralisation of a portion of the acid is more than compensated for by the increased stimulation which the gastric glands receive to produce hydrochloric acid. Sodium citrate, however, reduces the amount of hydrochloric acid available by converting it into sodium chloride. Barley water would seem to have no constant effect on the chemistry of digestion. Persistent vomiting in children is due to one of two causes—hypoacidity and hyperacidity. Test meals must therefore be given to such children to find out which condition is responsible for the vomiting. The most satisfactory test meal for infants is a five per cent. solution of lactose allowed to remain in the stomach for half an hour. A mixture of one part of milk with two of water may then be given to determine the degree of response to stimuli of which the gastric glands are possessed. Theoretically, when the acidity is below normal small doses of alkali or of hydrochloric acid should be given. When hyperacidity is present sodium citrate is the most likely drug to cause benefit. Protein digestion is slight in the infant's stomach, and is proportional to the quantity of hydrochloric acid present.

---

In the *Johns Hopkins Bulletin*, Berkeley reports the results of the experiments he has made with a view to determining the lesions produced in the nerve cells and vascular tissues by experimental Alcoholic Poisoning. He finds that it is the walls of the blood-vessels rather than the nerve elements which suffer from the effects of the drug in cases of acute alcoholic poisoning, and that the latter are involved more gradually than the mesoblastic tissues. Nevertheless, when administered in moderate doses over a long period, alcohol produces well-defined cell-changes in the nerves, as is shown by nuclear and dendritic alteration. Alcohol may be likened in its action on the nerve tissues to other poisons, such as ricin and the toxalbumins, but it has a much greater destructive effect upon the white cells as well as upon the cells of the blood-vessel walls. Moreover, the effect of the drug is proportionate to the amount administered, and also to the length of time which its poisonous action continues before the onset of death. If small quantities are given daily for a considerable period, they produce the same effect in a modified form as large doses administered for a limited period.

Dr. Giacomo Bonanno has published in the *Gazzetta degli Ospedali e delle Cliniche* the results of his researches into the action of morphia upon the resistance of red blood corpuscles. From experiments carried out upon dogs, he believes that injections of morphia diminish the resistance of erythrocytes. If, however, the blood is placed in contact with the alkaloid in vitro no diminution of resistance is noticeable. Moreover, this diminution is not the result of an accumulation of carbonic acid in the blood. It follows, therefore, that there must be some substances derived from the intracellular oxydation of morphia which, acting upon the blood, diminish the resisting powers of the red cells.

Drs. Lannelongue, Achard, and Gaillard have recently published an interesting little brochure, which gives a full account of their experimental researches into the influences which modify the evolution of tuberculosis. Their experiments were carried out on male guinea-pigs, divided into two lots of equal number and weights, one lot forming the subjects of the experiments, the other lot being used as controls. The following is a brief résumé of the results obtained by the authors. 1. Trauma. Twenty guinea-pigs were inoculated in the peritoneum with human tuberculous material, sputum, and pus. They were then subjected to various lesions such as fractures, separation of epiphyses, dislocations, articular contusions, etc. In no cases were tuberculous manifestations discovered at the site of injury, even when the lesion and the inoculation were made at the same time. The same result was noticed after repeating the experiments on rabbits inoculated with pure cultures of human tubercle bacilli. However, when large doses of tuberculous material were injected directly into the blood stream, tuberculous arthritis followed. Trauma, therefore, is not sufficient, by itself, to determine the migration of bacilli into an affected region. The principal cause of the localisation of a microbial infection would seem to be the nutritive activity of organs in process of development, a fact which may explain the frequency of osseous tuberculosis in children. Trauma to an articulation of a previously inoculated animal does not modify the evolution of osteo-arthritis, whereas normal movement carried out in an affected joint aggravates the symptoms and determines muscular contractures. Movement, therefore, hinders the cure of tuberculous swellings, the best treatment of which consists in complete immobilisation. (2) Climate would seem to have little influence on the evolution of the disease, for inoculated guinea-pigs lived longer in Paris than those sent into the country, to the seaside, and to the mountains. (3) Great and sudden changes of temperature accelerate the progress of the disease. (4) Inhalations of dust aggravate symptoms. (5) Animals made to take exercise die more quickly than those kept at rest, rapidity of death being proportional to the severity of the exercise. (6) A scanty diet shortens life. (7) Animals fed on glutinous food live longest; those fed on sugar outlive those fed on fat. (8) Alcohol shortens life; small doses frequently repeated are found to be more toxic than occasional large doses. (9) The progress of the disease is greatly modified by individual idiosyncrasy in animals which to ordinary tests of weight, size, etc., are exactly similar. (10) The authors have experimented with a serum derived from horses inoculated with a toxin derived from human bacilli. This was found to have properties which are wanting in normal serum. Preventive injections of this antitoxic serum have not been without effect, though no specific properties have been discovered. The authors are, therefore, hopeful that some specific remedy will before long be found for tuberculosis, though they are inclined to believe that treatment will have to be largely preventive rather than curative.

In the *St. Paul Medical Journal*, Dr. L. Dickinson discusses the uses of galvanism in the treatment of hæmorrhoids, fistulæ, prolapse, ulceration, and non-cancerous stricture of the rectum. When employing galvanism it is necessary to be cognisant of the particular properties of each pole. The positive pole gives off oxygen, is acid, hæmostatic, and sedative. It hardens the tissues, produces firm scars, and is vaso-constrictor. The negative pole gives off hydrogen, is alkaline, vaso-dilator, augments hæmorrhage, increases sensibility, softens the tissues, and forms soft scars. Hæmorrhoids can be treated with success by the electric needle introduced into the hæmorrhoid and connected with the positive pole. The negative pole is applied direct to the abdominal wall. A current of 15 milliampères should be passed for from 15 to 20 minutes. For anal fissure the positive pole should be applied to the lesion by means of a copper electrode. This should be left *in situ* until a deposit of oxychloride of copper is formed. Five or six applications are necessary to effect a cure. If the edges of the fissure are hypertrophied the negative pole should be applied to destroy the exuberant tissues. In rectal prolapse the positive pole is introduced into the rectum, and a current of 25 millampères passed through daily for a quarter of an hour. In stricture of the rectum an olive-shaped electrode, two or three times the size of the calibre of the stricture, is applied to the site of the lesion and connected with the negative pole. A current of from 15 to 20 milliampères is passed through until the tissues have become sufficiently relaxed to allow the electrode to enter the stricture. This treatment is continued for five or six days. If small ulcerations are present in the rectum, the cavity is first filled with normal saline and a long copper electrode introduced, which is in connection with the positive pole. A current of from 25 to 30 milliampères is used. This treatment is continued until the deposit of copper salt has produced its effects. Large and deep ulcerations are treated by the direct application of a mercury and zinc electrode, using a current of 20 to 30 milliampères.

